# Voices That Care: Licensed Practical Nurses and the Emotional Labour Underpinning Their Collaborative Interactions with Registered Nurses

**DOI:** 10.1155/2011/501790

**Published:** 2011-10-26

**Authors:** Truc Huynh, Marie Alderson, Michelle Nadon, Sylvia Kershaw-Rousseau

**Affiliations:** ^1^Faculty of Nursing, University of Montreal, Montreal, QC, Canada H3T 1A8; ^2^Jewish Rehabilitation Hospital, Laval, QC, Canada H7V 1B2

## Abstract

Recognizing the emotional labour underlying interprofessional collaborations (IPCs) could be considered a crucial step towards building a cohesive nursing team. Although IPCs between registered nurses (RNs) and licensed practical nurses (LPNs) have been linked to quality nursing care, little is known about the emotions experienced by LPNs during their interactions with RNs or those factors that influence IPCs. A questionnaire administered to 309 LPNs found that (1) the professional identity of LPNs has evolved into a that of a unique social group; (2) LPNs define IPC as an interpersonal process of exploring similar or dissimilar assessments of a patient's status with RNs and, together, establishing a course of nursing actions; (3) the primary organizational factor facilitating IPCs is inclusive nursing leadership; (4) the interpersonal factor promoting IPCs is the level of trust RNs extend to LPNs; and (5) an LPN's emotional labour (i.e., internal emotional regulation) is most tangible during uncollaborative interactions with RNs.

## 1. Introduction

In Canada and other developed nations, licensed nursing support staff are increasingly called upon to provide technical care (e.g., monitoring an taking vital signs, changing simple wound dressings) to society's most vulnerable populations (e.g., the elderly, the handicapped) [1]. They also offer relational care to patients: empathic listening and a personalized, caring dialogue, among others services [2, 3]. While licensed nursing-support staff have long predominated in long-term care settings, the number of licensed practical nurses^1^ (LPNs) working in acute-care institutions is on the rise due to persistent nursing shortages and the rationalization of nursing resources [4]. 

Licensed practical nurse (LPN) (in Quebec infirmier(ère) auxiliaire) and more rarely licensed vocational nurse (LVN) are the terms used in most states and provinces across North America to refer to a nursing professional who provides care for the sick, injured, convalescent, or disabled under the direction of a registered nurse. The Canadian province of Ontario also employs the term registered practical nurse (RPN). Outside of North America, the term enrolled nurse (EN) is used in Australia and New Zealand and state enrolled nurse (SEN) in the United Kingdom. LPNs work in a variety of settings: hospitals, residential care facilities, clinics, private homes, and doctors' offices. A certified LPN can provide a range of patient care dealing with routine nursing tasks (assisting patients to bathe, to go the bathroom, and in physical therapy) and patient monitoring (charting changes and collecting test samples). In Quebec, LPNs can also start “unmedicated” intravenous (IV) therapy, perform minor procedures, change dressings, and other similar tasks under the supervision of an RN or physician.

As the complexity of nursing care augments exponentially, along with the acuity of patients' medical disorders, the working relationship between registered nurses (RNs) and LPNs, specifically interprofessional collaboration (IPC),^2^ is a decisive factor in nursing care, one that significantly influences the quality of care [5–7]. Many factors intertwine to modulate IPCs within different systems: the professional system (different RN and LPN scopes of practice), the organizational system (leadership styles), the interpersonal system (RN-LPN interactions), and the personal system (personal values, attitudes, etc.) [8–11]. In this study, we aimed to address the organizational and interpersonal determinants of IPCs between RNs and LPNs in Quebec, a province of Canada as well as the professional factors affecting IPCs.

In the French-speaking province of Quebec, the professional body covering LPNs (infirmier(ère) auxiliaire), Ordre des Infirmières et Infirmiers Auxiliaires du Québec^3^ (OIIAQ: the Quebec order of licensed practical nurses), has a membership of more than 23,500. Registered nurses are covered under a separate association, Ordre des Inifmières et Infirmiers du Québec (OIIQ: The Quebec Order of Nurses). In 2003, with the adoption of Bill 90,^4^ the LPN's scope of practice, as well as that of the RN, was substantially expanded to facilitate greater LPN contribution to patient assessments and therapeutic nursing plans^5^ (TNP), more frequently referred to a nursing care plans outside of Quebec [12, 13]. 

Additionally, this new regulation, which resulted from collaborative initiatives between the professional associations (OIIQ and OIIAQ), authorized LPNs to undertake a greater role in clinical activities, such as, administering normal saline intravenous therapy and nursing care to respirator-dependent patients. In light of these major changes in the scope of practice, an in-depth look at RN-LPN collaborative interactions and their shared ownership over patient care and nursing interventions is imperative [14, 15]. 

The interpersonal system affecting RN-LPN collaborations is also important. In fact, RN-LPN collaborations at the bedside are essentially an interpersonal process: during daily interactions both professionals are emotionally engaged with each other, hence, both must regulate their emotions (e.g., trust, frustration) in order to work harmoniously for the benefit of their patients [16–18]. The process of regulating emotions has been conceptualized as “emotional labour” [19]. Within the field of organizational sociology and psychology, emotional labour has developed into a unique domain of research looking at the labour or the energy that workers expend to modulate their workplace emotions during encounters with coworkers, managers, and the recipients of their services [20, 21]. 

Nonetheless, within this domain, the emotional labour underpinning RN-LPN interactions has gone relatively unexplored in the nursing literature. According to Gray and Smith [17], emotional labour has not been extensively studied due to the fact that the emotions accompanying healthcare work are often silenced by the nursing staff themselves, in part, because of the espoused value of altruism inherent in nursing care.

Consequently, this study's originality resides in its description of the multiple factors that influence IPCs between RNs and LPNs and the relatively-undocumented emotional processes deployed by LPNs in their interactions with RNs. Study questions examined: (a) What is the nature of the RN-LPNs interactions? (b) What interpersonal and organisational factors facilitate or hinder collaborative interactions? (c) What is the emotional labour performed by LPNs during interactions with RNs? 

In the first part of this paper, a concise literature review of IPCs between RNs and LPNs is discussed within the context of the influencing factors of two systems in particular, organizational and interpersonal systems. The literature review concludes with selective readings focused on the emotional labours underpinning IPC. In the second part, the results of a “paper-and-pencil”^6^ questionnaire administered to 309 Quebec LPNs during their 2008 annual congress is presented and discussed.

## 2. Literature Review

### 2.1. Interprofessional Collaborations

The literature review revealed few findings concerning IPCs between RNs and LPNs. Miller and her colleagues [22] surmised that RNs and LPNs form a collegiality, a cohesive community of caring professionals, based on their professional identity and intergroup dynamics [23]. More significantly, Hallin and Danielson [24] argue that RNs perceive their work environment as stimulating when they are not constrained by workload and, consequently, have time to mentor licensed nursing-support staff towards optimal nursing care. Unruh (2003) also contends that RN-LPN collaborations lessen an RN's workload even with the additional task of LPN supervision [25]. 

Conversely, other scholars have noted discordant situations between RNs and LPNs [26]. Certainly, RNs and LPNs share common disciplinary approaches to care but at different levels, due to their differential educational backgrounds. Incompatibility between RNs and LPNs often takes root in role ambiguity, confusion related to the day-to-day application of their unique roles, and differential social status within the organizational hierarchy [27–30]. 

In a qualitative study examining RN-LPN collaborations in wound care, Huynh and Nadon [31] observed that RN-LPN collaborative interactions, at the microlevel, are influenced by mutual trust. Both professionals must manage their emotions (i.e., trust or distrust towards the other) while balancing respect for their colleague's professional role (i.e., RN or LPN) against an individualized trust for the professional as a person [31].

### 2.2. Factors Influencing Interprofessional Collaborations

As aforementioned, collaboration as an interpersonal process is influenced by multiple factors operating within several systems: organizational, professional, interpersonal, and personal. Based on the data from the literature, organizational factors that impact on IPCs include nursing leadership [32, 33] and organizational culture [34]. 

The first factor, effective nursing leadership, is said to exist when high-level nursing administrators and mid-level nurse managers encourage RNs and licensed support staff to communicate openly about the understandings and misunderstandings encountered during mundane care-giving activities, to engage in collective problem identification and resolution, and to strive continuously to build collaborative teamwork practices [33, 35, 36]. Moreover, inclusive nursing leadership is indicated by the consistent presence of nurse managers at the unit level, coupled with a demonstration of patience, empathetic listening and respect for the entrenched habits of the frontline nursing-support staff, as well as managerial skill in allocating equitable workloads among the nursing staff [6, 37–39]. 

Concurrent to this, in examining the second organizational factor, Upenieks and her colleagues [40] showed that an organizational culture which empowers nursing staff to initiate innovations in the delivery of nursing care (e.g., bedside nursing rounds similar to medical rounds) also leads to increased communication between nurses and nursing assistants. Similarly, an organization that promotes a culture of patient safety also witnesses optimal collaboration among frontline nursing staff (i.e., RNs, LPNs) [41]. In terms of institutional supports (i.e., the third organizational factor), well-established and accessible organizational structures (e.g., regular team meetings), and oft-used channels of communications (e.g., billboard for informal and formal memos) were found to contribute to interprofessional collaboration [42, 43].

Trust and respect, aspects emanating from the interpersonal system, have consistently emerged in the literature as the emotional factors most influencing the nature of the interactions among health professionals in general and, more specifically, between nurses and nursing support staff [37, 41, 42, 44–51]. Mutual trust within the nursing team is considered to be the cornerstone of IPCs. Based on this mutual trust, RNs and LPNs exchange patient information and feel empowered to actively seek out and provide constructive feedback to each other [52]. 

Respect, as an interpersonal factor, was found to be one of the key determinants of RN-LPN collaborative interactions [37, 53]. Respect is evidenced by the reciprocal recognition of coworkers' work experiences and competencies, and is accompanied by active listening, reflective comments, careful consideration, and empathic attention to the other person's inputs vis-à-vis a patient's health status [39, 46, 54]. 

This being said, within the context of overworked staff, due in large part to the chronic nursing shortage and prescribed organizational policies aimed at maximal productivity, the hectic pace of day-to-day nursing care does not lend itself to the fostering of the positive emotions critical for IPC: trust and respect [55]. Yet incongruously, RNs and LPNs are required to display these unfelt emotions towards their colleagues [18]. The invisible emotional labour of having to display appropriate workplace emotions (e.g., respect, trust) while suppressing negative emotions (e.g., anger, frustration) may contribute to emotional and cognitive dissonance [17, 48, 56, 57] and, potentially, emotional exhaustion and burnout [58–62].

### 2.3. Emotional Labour

Emotional management in the workplace or, more specifically, emotional labour was first theorized by Hochschild [19]. “Emotional labour” refers to workers' expenditure of energy in suppressing and/or substantially changing their emotions, in compliance with organizationally defined rules and regulations, in order to display feelings that convey to others a sense of being cared for [63, 64]. As an internal personal process, emotional labour is conceptually distinct from the concept of emotional support, which is provided to patients as an essential and fundamental element of nursing care [65]. 

Within the nursing literature, emotional labour is comprised of two processes: the suppression of negative emotions (e.g., frustration, anger) and the expression of unfelt emotions (e.g., respect, trust) that are deemed to be workplace appropriate [17]. At present, there is a dearth of knowledge about healthcare workers' and the emotional labours inherent in collaborative endeavours with colleagues: particularly, those experienced by staff in lower hierarchical positions [16, 22].

## 3. Methods

During the 2008 OIIAQ annual congress, a survey entitled “The winning experience of collaboration between RNs and LPNs” was administered, with technical support and assistance from the OIIQ, to 309 LPNs. The survey's design employed convenience sampling, a nonprobability sampling technique [66]; no incentive was offered to the LPNs in exchange for their participation. However, different outreach strategies were utilized to maximize the response rate: regular intercom messages throughout the congress reminded the LPNs to complete the survey and researchers collected the questionnaires at the door. 

 The questionnaire was developed based on a literature review that focused specifically on the emotions experienced by nursing support staff during their interactions with colleagues and nurse supervisors [53, 67–69]. Findings from three studies suggest that during a delegation process,^7^ nursing support staff experience a multitude of conflicting emotions, negative and positive, that affect the nature of their future interactions with the RN in question. 

Thus, the researchers worked collectively on the wording of the questionnaire to ensure clarity and consistency based on the systematic application of an “Instrument Appraisal List,” as defined by Fowler and Cosenza [70]. Due to time constraints, field testing the questionnaire was not possible. The questionnaire consisted of 5 questions querying LPNs on their experiences of noncollaborative and collaborative interactions with RNs, their emotions resulting from these interactions, and their perceptions regarding the interactions of the RN. The LPNs were given the choice of selecting from a list of answers that fit their thoughts or of developing their own responses—an open and closed format questionnaire. (A copy of the questionnaire can be obtained by contacting the lead author).

## 4. Data Analysis

The response rate was 86%: 267 out of 309 LPNs completed the questionnaire. Data analysis comprised two phases: quantitative and qualitative. The first, the quantitative phase, served to compile and document the frequency of similar responses. In the second, the qualitative phase, ATLAS.ti software was used to manage and analyze the data. 

**Figure 1:**
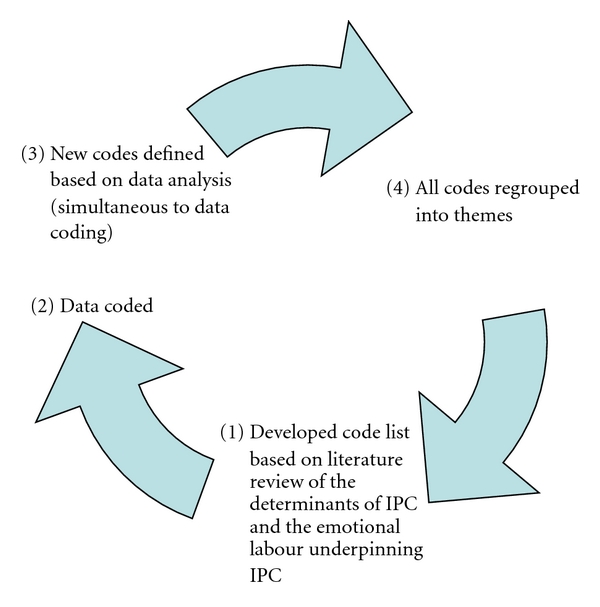
Steps in data analysis and data interpretation.

Data interpretation was undertaken using an initial list of template codes determined by the research team but based on the literature review [71]; for example, template codes included determinants (interpersonal, organizational) and emotions (negative and positive), and so forth. New codes that emerged during the coding process were noted by the individual researchers; inclusion of new codes was then discussed among the team members. Consensus was required in order to retain or discard new codes. Some of the new codes that were retained included auto-determination and pride, which were subsequently collapsed into the emergent theme of professional identity. Data ambiguity (i.e., where text segments were coded differently) was also debated in order to reach a consensus. Once completed, the codes sharing common elements were merged to form the following themes: factors contributing to IPCs, professional identity, and the emotional labour underpinning LPN-RN interactions (see Figure 1). An audit trail was kept of the rational for collapsing specific codes into themes and of the selected citations used to constructing the themes, see Table 1.

The internal validity of this study relied on the triangulation of qualitative and quantitative data. For example, quantitative data indicated that 80 per cent of LPNs believe that their interactions with RNs are of a collaborative nature; this concurred with the qualitative data, in which the same LPNs had described the collaborative interactions step-by-step. Triangulation among researchers, coders, and data was also undertaken. The lead author and the research assistant independently coded all the LPNs' written answers. The intercoder reliability was 85 per cent. Member checking was done by validating the preliminary results of the survey with the same population of LPNs who had completed the questionnaire.

## 5. Finding

### 5.1. Quantitative Results

A majority of study participants, 80 per cent (*n*: 214 of responding LPNs out of 267), confirmed that they collaborated with RNs daily; only 14 per cent indicated no collaboration. A further 5 per cent stated they had limited interaction with RNs. A few (*n*: 3 LPNs) indicated collaborating with specific RNs but not others; in terms of the latter, this was attributed to a lack of experience on the part of the RN—generally a young newly graduated RN. 

The primary interpersonal factor promoting IPC, as indicated by 63 per cent of the LPN respondents, was an RN's respect for their patient assessments. Another positive factor preceding IPC, as perceived by 28 per cent, was RN's actions in terms of the solicitation of and active listening to their assessment of a patient's status. Conversely, 62 per cent pointed out that the tension between them and RNs was rooted, in the LPN's perception, to an inappropriate volume of delegated acts (i.e., too much or too little). Moreover, the nature of the delegated acts, when viewed as “dirty work” (e.g., changing soiled undergarments), was also perceived to inhibit future collaborative interactions. 

Although the compilation of LPN answers did not yield a dominant trend, the most common organizational factors facilitating IPCs were leadership style, teamwork (as the primary care delivery mode), and, finally, the equitable distribution of workload among both RNs and LPNs. Indeed a significant percentage (40 per cent) of LPNs attributed a positive work environment, one facilitating IPCs, to the inclusive and compassionate leadership exemplified by the head nurses or their assistants; these leaders served not only as a source of professional and personal support but also as role models for collaborative interactions. Unsurprisingly, 16 per cent attributed their limited RN-LPN communications to an organizational factor: time constraints.

The questionnaire asked LPNs to describe their emotions following collaborative or uncollaborative interactions with RNs. After a collaborative interaction, the majority of the LPNs (68 per cent) felt valued by their nursing colleagues, 23 per cent expressed happiness, and 4 per cent indicated feelings of professional accomplishment and personal satisfaction. On the other hand, after uncollaborative interactions with RNs, 60 per cent of the LPNs felt frustrated, 25 per cent discouraged, and 2 per cent saddened, and 1 per cent expressed aversion to any further interaction with the RN in question. While 1 per cent of the LPNs chose to distance themselves emotionally (i.e., indifference) to uncollaborative interactions with an RN, 3 per cent decided to be affirmative: attempted to readdress the issue with the RN or brought the matter to the attention of the nurse manager or the union representative.

### 5.2. Qualitative Findings

This section examines the themes that emerged from an analysis of the descriptive responses: the organizational and interpersonal factors facilitating the collaboration, professional identity, and emotional labour that underlie collaborative and uncollaborative interactions. These responses explicate, in depth, the LPNs' perspectives.

#### 5.2.1. Organizational Facilitator of Interprofessional Collaboration

Most of the LPNs defined IPC as an interpersonal process of exploring similar or dissimilar assessments of a patient's status with an RN and, together, formulating a course of nursing actions. One LPN emphasized: “The perfect and concrete example of collaboration that I can give is when the nurse shares my own ideal of wound care, has the same motivation like me to speed up wound healing; she follows up on my suggestion about changing wound treatment, she revises with me the wound care plan.”

Inherent in this definition is that IPC, in the delivery of daily nursing-care activities and in terms of equality between RNs and LPNs, is founded on the concept of “togetherness”. The following quotes also attest to how these two organizational factors (i.e., togetherness and equality) facilitate IPC. “In our palliative unit, I work on an equal basis with the nurse: she has 4 patients so do I; we help each other in keeping an eye on the other's patients.” and “I work closely with the nurse in my paediatric unit, one day she gives the medication to the patients, I take the vital signs, the next day, I give the medications and she takes the vital signs, this way we get to know all our young patients.” 

Other LPNs also believed that collaboration takes place when LPNs are able to share their technical and experiential knowledge (i.e., the unit work routine), as an equal, with newly-graduated RNs. Another organizational factor that facilitates not only IPC but also the professional development of LPNs is an organizational culture that promotes innovative actions; as exemplified by this quote: “On our unit the LPNs are invited by nurses to discuss their patients during team meetings with the doctors and other professionals. They encourage us to try out new initiatives that help the patients and we can become more efficient.” 

The most prevalent organizational factor was nursing leadership. An RN's leadership style was deemed to be inclusive when RNs explained the rational underlying nursing interventions, encouraged the LPNs to initiate innovative actions, and showed their appreciation or work completed. In contrast, the LPNs affirmed that an organizational culture promoting individual work impedes collaborative interactions with RNs.

#### 5.2.2. Interpersonal Facilitator of Interprofessional Collaboration

The most prevalent interpersonal factor expediting IPC was the level of trust RNs exhibited towards LPNs, as depicted by one LPN: “The young RNs often ask for our opinions, they trust our judgement and we are happy to be their eyes to assess the patients for them.”

The second most common interpersonal factor mentioned by the LPNs was the respect conveyed by RNs towards LPNs by means of compliments, as illustrated by this quote: “Teamwork is also reciprocal respect as when the nurse makes the effort to thank me for taking initiatives in her absence such as one time; I kept a closer eye on the resident and reported his worsening status to the doctor.”

#### 5.2.3. Professional Identity

Written narratives from the LPNs described their workplace self-identity as that of a professional, as evidenced by this quote: “I have my own professional license; I always substantiate my nursing actions with my nursing knowledge, I know that I have to be always accountable for every action that I undertook.”

At the interpersonal micro level, LPNs believe that their contribution to the nursing team is significant. One LPN stated:

“As a health worker, sometimes, I feel at loss at all the suffering around because there are a lot of patients being abandoned by family members to die, but being an LPN and a member of my strong nursing team, I got a chance to offer some last comfort to one dying patient and also because the other RNs help me with my other chores; that day I walked out of the hospital prouder thanks to my team, “my nurses” who really recognized my value as an LPN.”

This professional self-identity was strengthened even further with the advent and clinical application of Bill 90, the professional law that expanded the LPN's scope of practice. A large proportion of the LPNs' written answers denoted professional pride in their work: assessing wound status, discussing with RNs, and contributing to the elaboration of wound-treatment plans as well as to a positive outcome (i.e., wound healing). Other LPNs indicated that these newly acquired skills in wound care added to their sense of personal satisfaction at work.

On the other hand, LPNs' increased professional identity as a social group, distinct from RNs, has had some unintended effects; such as the unconscious application of the social distancing strategy, often using negativism to distinguish themselves as a group distinctive from RNs, as demonstrated by the following quote: “Some nurses do not care what I say to them about the patients; they just like to sit and chat all day at the nursing station among themselves.”

#### 5.2.4. Emotional Labour

Several LPNs described the intense but internal, thus invisible, effort they had exerted in managing their emotions (i.e., to perform emotional labour) in order to suppress negative feelings following unproductive encounters with RNs. Surprisingly, the LPNs rarely indicated employing the strategy of surface acting (i.e., expressing an unfelt emotion of respect) during instances of disagreement with an RN. Conversely, some of the LPNs' negative remarks about RNs not only served to differentiate themselves from RNs as a group, but also to suppress (by redirecting) their feelings of being devalued and belittled after uncollaborative interactions. One LPN eloquently stated: “I reported repeatedly to the nurse that the patient's status is getting worse, still nothing was done; it was as if my voice is inaudible, my assessment and me as LPN are worth nothing.”

Yet another LPN revealed her emotional labour: “One time, one young and inexperienced nurse refused to believe that the patient's in a lot of pain, I had to keep my calm and discussed patiently with her again and again before she agreed to medicate the patient.”

An analysis of the numerous accounts of emotional labour experienced by the LPNs during IPCs underscored the fact that LPNs who possess a strong professional identity, accumulated as a result of many years of practice, are more likely to regulate their emotions in order to transcend conflicts with RNs. One LPN argued with dignity and passion:

“Working on this unit for a long time, I have made a few mistakes but I also have a lot of experience as an LPN, a professional; so sometimes I refuse to take “no” from the nurse. She has more years at school than me but I think that when we talk rationally together we can come to an agreement calmly for the sake of our patient and not to see whether me or she is right.”

## 6. Discussion

The authors recognise that the study has several limitations. The first emanates from the fact that a self-report method, as used in this study, means the results are subject to a positive self-representation bias and limited by one-sided view: the LPN perspective. The title of the questionnaire, “*The winning experience of collaboration between RNs and LPNs”*, might have inadvertently directed the LPNs to view RN-LPN collaborations in positive terms, hence have influenced their responses. Nonetheless, one of the questions invited LPNs to describe uncollaborative interactions with RNs, to delineate the determinants of uncollaborative interactions and the impact on their emotions. Finally, the fact that the questionnaire was not field tested could imply a reduced confidence in its face validity and quantitative findings. However, as mentioned previously, this risk was mitigated by having the research team carry out triangulation of both quantitative and qualitative findings to substantiate emergent themes. 

The study has given rise to several interesting findings. The first of which responds positively to our first research question: RN-LPN interactions are of a collaborative nature. This fact, or least as the intended goal of the Ordre des Infirmières et Infirmiers Auxiliaires du Québec, was corroborated by a statement made by the OIIAQ's president in an open letter to Quebec's general population [72]. In the spirit of encouraging a continuous and constructive dialogue between RNs and LPNs, below, we discuss two of the main IPC-facilitating factors and the emotional labour underpinning uncollaborative interactions emanating from this study.

### 6.1. Factors Facilitating Interprofessional Collaboration

Inclusive nursing leadership was found, in this study, to be the main organizational factor facilitating IPC. In their study of teamwork in nursing homes, Wicke et al. [7] found that the junior nurse managers believe that the accessibility of the “matron” (the equivalent of senior-level nurse manager) in dealing with any management problems was vital to efficient and cohesive teamwork. This is also consistent with the present literature, in that, good nursing leadership at the executive and unit levels is seen to contribute to the maintenance of an organizational culture that is empowering to nursing staff [73]. The LPNs in this study also emphasized equitable workload-distribution as an organizational factor that enhances IPCs. This notion of equity does not mean that RNs and LPNs should have the same number of patients (as mentioned by one LPN in the previous section). According to several LPN-participants, and affirmed by Vogelsmeier's findings [33], equity or fairness constitutes an essential aspect of inclusive leadership—defined as one in which workload is distributed according to each individual's (RN and LPN) professional competences and in which feedback on this issue (workload) is valued.

In contrast with Kenney's [74] findings that trust is the outcome of RN-LPN interactions, the LPN-participants asserted that trust is itself the most crucial interpersonal factor *leading to* rather than an outcome of IPC. In concordance with other studies, the LPNs affirmed that trust is personalized: the LPNs trusted some RNs and not others based on past experiences with the RN in question and their perceptions of the RN's job-related competencies [46, 75].

### 6.2. Emotional Labour Underpinning IPC

The literature is replete with studies that look at workplace conflicts among nursing staff and between nurses and other health professionals through the lens of communication theories [76, 77]; parallel to this, emotional labour represents an alternative theoretical perspective, one that brings to the forefront the invisible emotional work that the nursing staff (i.e., RNs and LPNs) undertake in their daily interactions with nursing colleagues and other health professionals. In fact, our findings point to the fact that IPC has two facets: instrumental and emotional. Instrumental IPC is the clinical application of the 5R Principles: right LPN, right task, right circumstance (i.e., does the complexity of task match the competency of the LPN and the availability of bedside clinical teaching), right communication or directives, and right supervision as specified by the relevant professional body [78]. Emotional IPC is the process whereby RNs respectfully entrust nursing interventions to LPNs and empowering them to discuss any disagreements in patient assessments and nursing care plans.

Supporting this, a small body of literature has consistently documented the close link between a worker's inferior social status, relative to the RN's, with the performance of emotional labour as a means of preserving dignity in the workplace [64, 79]. This study illuminates the emotional labour underpinning IPCs between RNs and LPNs. From the standpoint of a lower hierarchical status in the workplace, the LPNs in this study called attention to the emotional labour underlining their interactions with RNs; this is not dissimilar to the phenomenon whereby nurses manage their emotions in conflicting interactions with physicians [80]. Two further issues worthy of mention include the perspective, as expressed by the LPNs, that the LPN and RN roles are interchangeable (as illustrated by the first two quotes in Section 5.2.1) and the fact that the majority of LPNs are women. 

Although, from the LPN perspectives, some nursing activities such as taking vital signs and administering medication, and so forth, can be performed by either the LPN or RN, the global assessment of a patient's status (i.e., determining whether a patient needs any further medical interventions from a nursing perspective) remains the ultimate responsibility of the RN, as aptly illustrated by the quote “the LPN are the eyes of the RN.” 

The predominantly female character of the LPN profession and the fact that emotional labour is perceived to be intrinsically female, a fact many women are conscious of, may in fact contribute to a desire to suppress emotional labour, thereby contributing to its invisibility. Further, resignation at having little influence with which to challenge the existing social order, a status differential associated with asymmetrical power relationship, may also contribute to the invisibility of emotional labour [81].

## 7. Conclusion

It is not our intention to present an overidealized image of LPNs, but to recognise their working conditions and to identify factors that could improve the working environment. Historically, LPNs have been beleaguered with low status within the hierarchy of healthcare organizations [28]; consequently, the voicing of their experiences as a distinctive group of healthcare professionals is rarely heard. Concurrent to this, and within the context of the rising cost of health care, chronic shortages in the RN workforce, and the expansion of the LPN's scope of practice (as well as that of RNs), LPNs have emerged as a distinct social group in their interactions with RNs [79, 82]. 

In choosing to look at the emotional aspects of RN-LPN interactions, this study highlights the invisible emotional labours experienced by LPNs and the role of nursing leadership as it contributes to IPCs. This is even more pertinent, particularly to administrators of medical facilities, when juxtaposed with the findings of a large study by Duffield et al. [83]. The study examined the roles of RNs, LPNs, nurse managers, and clinical nurse specialists in 80 medical and surgical units and demonstrates that nurse managers face many great and concurrent challenges, such as, providing a supportive work environment for newly hired staff while simultaneously facilitating professional autonomy for all levels of nursing personnel, among a number of other challenges. Indeed, nurse managers are called upon not only to empower (i.e., guide, coach) nursing staff to carry out nursing care activities (e.g., wound care) to the highest standard, in accordance with their competencies and knowledge [84, 85], but also to recognize the importance of the emotional labour performed by their staff during encounters with colleagues and patients. 

Building on the findings of this study, further research is required to better understand the perspectives of both RNs and LPNs in terms of their collaborative and noncollaborative interactions and the emotional labour underpinning these interactions. Quasiexperimental studies designed to examine the effects of in-house training programs, that encompasse emotional labour, the frequency and quality of RN-LPN collaborative interactions, and, ultimately, the quality of patient care, are also required.

At the educational policy level, the authors recommend that emotional labour as an important concept, albeit an ill-understood one, be integrated into the curriculum of prelicensure RN and LPN programmes.

##  Ethical Considerations

The OIIAQ reviewed the questions, created the questionnaire's graphical design, and authorized its distribution to the LPNs attending its annual 2008 congress. All participants were assured of confidentiality, which was further reinforced by the anonymity of the questionnaire.

## Figures and Tables

**Table 1:** Template codes, emergent codes and themes.

Template codes	Emergent codes	Themes
*Characteristics of LPN*: years of experience, area of practice, self-identity as LPN	collective identity, auto-determination, pride	Professional identity
*Organizational factors: * inclusive leadership, directive leadership, teamwork, organisational culture, equitable distribution of workload	innovative actions, “togetherness", equality	Organizational facilitator of IPC
*Interpersonal factors: * respect, trust		Interpersonal facilitator of IPC
*Emotion: * anger, frustration	Feelings of being devalued, indifference	Emotional labour

## Endnotes

1. In Quebec, LPNs must successfully complete 1800 hours (the equivalent of one year) of training to obtain an LPN diploma from the Ministère de l'Édcation, du Loisir et du Sport du Québec (Quebec Ministry of Education, Recreation and Sport). The primary difference between Quebec RNs and LPNs is that RNs are required to elaborate Nursing Therapeutic Plans (Plan Thérapeutique Infirmier) based on patient assessments and to adjust according to a patient's evolving status while LPNs contribute to the implementation and modification of these plans. In other words, LPNs report and discuss their observation of a patient's status with the RNs and suggest changes based on their observations.
2. In most North American and European countries, RN and LPN collaboration is deemed to be intraprofessional. However, in Quebec, Canada, given the fact that the RNs and the LPNs belong to two distinct professional bodies, the term interprofessional collaboration (IPC) is utilized to describe their workplace interactions.
3. The official language of Quebec is French, thus the professional orders of healthcare professionals (such as registered nurses and licensed practical nurses) retain their French titles even in documents addressed to their English-speaking members and the general public.
4. The Quebec government introduced and adopted Bill 90 in order to modernise the scope of practice of several health professionals, medical doctors, registered nurses, and licensed practical nurses- within the spirit of facilitating interprofessional collaborations among health professionals.
5. The therapeutic nursing plan, an application of Bill 90 (art. 36), defines the scope of nursing practice and includes three activities legally attributed to nurses: (1) assessing the physical and mental condition of a symptomatic person, (2) providing clinical monitoring of the condition of persons, whose state of health is problematic, including monitoring and adjusting the therapeutic nursing plan, and (3) providing nursing-care followup for persons with complex health problems.
6. As defined by Foddy, W. H. (1994). Constructing questions for interviews and questionnaires: Theory and practice in social research (New ed.). Cambridge, UK: Cambridge University Press.
7. Delegation is the assignment of authority and responsibility to another person to carry out specific activities.
